# A recurring rollercoaster ride: a qualitative study of the emotional experiences of parents of children with juvenile idiopathic arthritis

**DOI:** 10.1186/s12969-016-0073-9

**Published:** 2016-03-09

**Authors:** Oralia Gómez-Ramírez, Michele Gibbon, Roberta Berard, Roman Jurencak, Jayne Green, Lori Tucker, Natalie Shiff, Jaime Guzman

**Affiliations:** Department of Anthropology, University of British Columbia, 6303 NW Marine Drive, Vancouver, British Columbia V6T 1Z1 Canada; Division of Rheumatology, Children’s Hospital of Eastern Ontario, 401 Smyth Road, Ottawa, Ontario K1H 8 L1 Canada; Department of Pediatrics, Western University and Children’s Hospital, London Health Sciences Centre, 800 Commissioners Road East, London, Ontario N6A 5 W9 Canada; Department of Pediatrics, University of Ottawa and Children’s Hospital of Eastern Ontario, 401 Smyth Road, Ottawa, Ontario K1H 8 L1 Canada; British Columbia Children’s Hospital, Room K4-116, 4480 Oak Street, Vancouver, British Columbia V6H 3V4 Canada; Department of Pediatrics, University of British Columbia and British Columbia Children’s Hospital, 4480 Oak Street, Vancouver, British Columbia V6H 3V4 Canada; Department of Pediatrics, University of Florida, and Department of Community Health and Epidemiology, University of Saskatchewan, 1600 Archer Road, Gainesville, Florida USA

**Keywords:** Juvenile arthritis, Emotions, Parents, Canada, Qualitative research, Secondary analysis

## Abstract

**Background:**

Despite the wealth of clinical research carried out in children with juvenile idiopathic arthritis (JIA), little is known about the emotional experiences of their parents. This article describes the predominant emotional experiences reported by parents of children with JIA in two Canadian cities.

**Methods:**

Research participants included 15 experienced parents and 8 novice parents (<6 months since children’s JIA diagnosis). Their children were 2 to 16 years old with various JIA categories. A qualitative dataset including audio recordings and verbatim transcripts of three focus groups, and written reports of 59 reciprocal interviews (parents interviewing each other) were examined by a multidisciplinary research team following a four-step qualitative analytical process.

**Results:**

Parents of children with JIA experienced recurrent mixed negative and positive emotions that varied over time. Between disease onset and diagnosis, mounting anxiety, fear and confusion were the predominant emotions. Shortly after diagnosis there were shock, disbelief, and fear, with a sense of having being blindsided by the disease. At times of disease quiescence there was hope and gratitude, but also fatigue and frustration with ongoing treatment and fear of flares. During periods of increasing or ongoing symptoms there was admiration and sympathy for the courageous way children coped with JIA, as well as sorrow and frustration for ongoing pain and limitations. There were at times, frustration and indignation with peers and teachers unable to understand the child’s fluctuations in physical activity and schoolwork. Throughout the disease, parents felt an underlying anxiety and powerlessness.

**Conclusions:**

Parents of children with JIA described complex emotional journeys akin to the recurring ups and downs of rollercoaster rides, instead of ordered emotional phases ending in resolution. This has implications for healthcare providers who need to be aware of the complexity of these emotional journeys to support parents more effectively, thereby helping improve patient outcomes.

## Background

The diagnosis of a chronic illness in a child is one of the most emotionally difficult situations a parent may face [1, 2]. Despite the wealth of clinical research carried out in children with JIA [3–7], little is known about the emotional experiences of their parents. Published research reports strong correlations between parental experiences and perceptions and juvenile arthritis outcomes [8, 9]. Research in other childhood conditions supports a connection between parents’ illness-related beliefs and the fostering or hampering of children’s health practices [10–12]. A link between parental emotional experiences and clinical outcomes has been suggested in research in children with anorexia, cleft palate, autism, and cancer [13–16]. Since parental experiences in a number of chronic health conditions seem to be connected to disease outcomes, understanding parental experiences in JIA may be illuminating for care providers interested in optimizing clinical outcomes for children with this disease, and ultimately all rheumatic diseases.

During a previous qualitative study we conducted to identify clinical features of the disease that were most important for patients, parents and clinicians in the course of JIA [17], we were confronted with parents’ intense emotional experiences about the disease. Qualitative research emphasizes being willing to listen and pay attention to what research participants identify as important, even when it departs from the original research aims [18]. Upon consideration of these parental emotions, we felt that a focused description of these emotional experiences was owed to study participants who wanted their stories heard; and that such description would be instructive to health care providers caring for children with JIA and their families. We thus decided to pursue a secondary analysis of the qualitative data set collected in our previous study to address two new research questions: (1) What are the predominant emotional experiences of parents caring for a child with JIA? (2) How are these parental emotions associated with different phases or events during the disease?

McNeill has described the emotional challenges fathers of children with JIA face in dealing with their child’s pain, variability in the severity of the condition, adherence to the treatment regime, and uncertain prognosis [19]. The present study adds to McNeill’s findings by showing that the emotional experiences of mothers and fathers of children with JIA vary throughout the course of the illness in complex emotional journeys akin to the ups and downs of rollercoaster rides, related to the social and medical challenges of managing a child with the disease.

## Methods

This study presents secondary analysis of an existing qualitative dataset [20, 21]. Our original project sought to prioritize clinical features of the disease course of JIA according to patients, parents and clinicians through six study sessions [17]. Here we report on the three study sessions that included parents of children with JIA. Each of these three sessions included a focus group and a series of reciprocal interviews (participants interview each other) [22]. The sessions were carried out in two Canadian cities between July and November of 2012 with participation of 23 parents. The first study session was held in English with 10 experienced parents; the second session was held in French with 5 experienced parents; and the third session was held in English with 8 novice parents (<6 months since diagnosis). The dataset comprised audio-recordings and verbatim transcripts of the three focus groups, as well as structured hand-written reports provided by the parents after 59 reciprocal interviews.

Recruitment occurred during routine clinic visits in two Canadian academic pediatric hospitals. Fathers and mothers were approached for participation, and couples were welcomed to participate. We purposefully included parents of children with different JIA categories, various lengths of experience in dealing with the disease, and English or French cultural/linguistic backgrounds (Canada’s official languages). Our goal was to have representation of a wide range of disease experiences, but we did not aim for proportional representation of the general population of children with JIA. Our original design called for recruitment of experienced parents (>2 years since diagnosis) and novice parents (<6 months since diagnosis), but when a parent with 9 months of experience volunteered to participate in the study s/he was included with the experienced group. During analysis for this study (see below), other background characteristics such as the gender of the parent, age of the child and previous family experience with arthritis were considered for their relation to expressed emotions.

Focus groups started with a round of introductions where each parent shared the salient aspects of their experience regarding their child’s JIA, before moving onto prioritization of features of the disease course. Each parent participated in 2-to-3 reciprocal interviews (as time allowed), taking the dual roles of interviewee and interviewer. We believe this opportunity for one-on-one exchanges with other parents helped create an environment more conducive to the expression of emotions, and that the written reports provided a chance for reflection and expression of what they had heard in their own words [22, 23].

We obtained ethics approval for both the primary and secondary studies from the research ethics boards at British Columbia Children’s Hospital and the Children’s Hospital of Eastern Ontario.

### Analysis

We followed a four step qualitative analytical process [24, 25]. Analysis was done using word processors and spreadsheets, instead of software specially designed for qualitative research. Since the expression of emotions is highly dependent on the context, and emotions are often manifested by non-text cues such as pauses, changes in voice intonation, body language, crying, laughing, etc., we listened to the original audiotapes, and when necessary, relied on the accounts of people who were present during the original study sessions. Details of the four steps are described below.

First, OGR listened to the original focus group audio-recordings several times. She added to the existing verbatim transcripts extra annotations that could inform coding of emotions, such as pauses, utterances, changes in voice intonation and speed of speech, crying, laughs and background noises [26]. She had not been present during the original focus groups but when in doubt she consulted with MG and LT, who were present. Written reports of reciprocal interviews did not need further preparation as they had already been entered in spreadsheet files.

Second, two researchers (OGR, MG) carried out initial coding of emotions assisted by a bilingual list of 69 potential emotion labels derived from pertinent literature. The list was based primarily on the work of Plutchik [27] and Kübler-Ross [28]. A directed content analysis approach [29], was used to code every instance of emotional experience present in the datasets. Four researchers (OGR, MG, JGr, JGu) held weekly conference calls to provide feedback during coding, including refinements and additions to the initial list of emotion labels.

Third, two investigators (RB, RJ) carried out coding verification through independent assessment of the initial coded files. This internal review helped confirm that all emotional instances had been flagged and that the assigned emotion labels were accurate [30]. When in doubt about an emotion label, we based our final coding decision on audio cues, the reports prepared by the professional facilitators of the study sessions, and the interpretations of those in our research team (MG, LT) who had been present during the sessions.

Fourth, all investigators in this study examined and reflected on the coded files individually, and then discussed them collectively during conference calls. One conference call was devoted to each of the three sessions. During this reflection and discussion we asked what emotions were predominant at different stages of the disease, who or what were those emotions directed at, and how the background of participants shaped their emotional experiences. Background was broadly interpreted to include gender, language, length of experience with the disease, child’s JIA category, and previous experience with another rheumatic disease.

## Results

Table 1 shows the characteristics of the 23 study participants. Participants represented a wide range of parental length of experience with the disease: 15 were experienced parents with 9 months to 14 years of experience caring for a child with JIA, and 8 were novice parents within 6 months of the diagnosis of JIA. Their children were 2 to 16 years of age and had a variety of JIA categories and disease severity.

**Table 1:** Characteristics of study participants

Participants	Characteristics
Experienced Parents	15 parents (8 mothers, 7 fathers, including 3 couples).
	Their 12 children (9 girls, 3 boys) were 4 to 15 years old.
	Children had been diagnosed with JIA 9 months to 14 years prior to the session.
	8 children had oligoarthritis, and 1 each had enthesitis-related arthritis, psoriatic arthritis, polyarthritis, or undifferentiated arthritis.
	One parent had JIA as a child, another had psoriasis, and one more had ankylosing spondylitis. A parent’s spouse (not in attendance in the session) had JIA as a child requiring two hip replacements.
Novice Parents	8 parents (5 mothers, 3 fathers, including one couple).
	Their 7 children (4 girls, 3 boys) were 2 to 16 years old.
	Children had been diagnosed with JIA 2 to 6 months prior to the session.
	2 children had oligoarthritis, 2 had undifferentiated arthritis, and 1 each had systemic arthritis, enthesitis-related arthritis, or psoriatic arthritis.
	One parent had another child with Crohn’s-related arthritis. A parent’s spouse (not in attendance in the session) dealt with osteoarthritis and two knee replacement surgeries.

*JIA* juvenile idiopathic arthritis

### Period leading up to diagnosis

Parents recounted the period between symptom onset and diagnosis as a time of mounting anxiety, fear, and confusion. Similar emotions were reported by parents regardless of their specific circumstances and length of familiarity or experience with JIA. Many parents said they had initially thought their children merely had an injury. The intensity of their mixed feelings of anxiety, fear, and confusion was shaped by the relative ease or difficulty in arriving at a diagnosis. For some parents, this was also a time punctuated by frustration with the health care system as families experienced prolonged waits to be seen by a paediatric rheumatologist with ensuing delay in diagnosis. Other parents expressed indignation about having being ignored, belittled, or even suspected of child abuse due to their child’s unexplained joint swelling. This was perceived as further delaying referral to the specialist and arrival at the diagnosis. Table 2 shows illustrative quotes from parents.

**Table 2:** Quotes from parents that illustrate the emotions experienced at different stages of the disease

Disease stage & predominant emotions	Illustrative quotes
Period Leading Up to Diagnosis	
confusion, anxiety	
	And now all of a sudden he started complaining about his hip, and you know, he could have been… He’s a boy, so I was hoping that it was just that. But he had some inflammation, and he complained, so I was concerned. (EF)
frustration	
	In my case, she [daughter] had a streptococcal throat infection, which was treated with antibiotics, and then it started to swell, and then she started… She started limping. I went to the doctor two more times, and I said, “Listen, something’s not right. When she wakes up, when I go to get her from her crib, it’s still swollen, and her knee is bent, and she can’t straighten her leg for a good two hours.” … I wasn’t getting any response from my family doctor… [so] I went to [hospital name] and insisted on an appointment with a rheumatologist, and that she [daughter] be seen within three days. (EM)
indignation, fear	
	It’s rather interesting, right from the get go: “You’re going to have to bring the child into emergency.” Okay…I flew in from [another city] and met my wife. Off to the hospital we went… And this was after my wife was interrogated the evening before, wondering if she was beating the child or what the case may be, because her ankle was just swollen out like a… like a baseball. And for a two-year-old child that’s quite traumatic, quite frankly. (EF)
Upon and Shortly After Diagnosis	
shock, disbelief, fear	
	No, no signs, no instances. It’s funny, I’m getting emotional, the first time talking about it. (broken voice, brief pause) So we didn’t have… We didn’t actually get any documentation or introduction to the fact that it might be juvenile arthritis. We thought it was an injury. So we had done peripheral research, (broken voice) but didn’t really know… Sorry, I need to back away for a second. (silence) (EF)
shock, disbelief, surprise	
	Just (brief pause) shocked I guess when I heard that she had arthritis, simply because I’d never heard of a child with arthritis before… And then coming here and realizing that it’s more common than people are [aware of] or than it’s known to be to people, I guess. (NF)
fear, sorrow	
	“Oh my God, what’s going to happen to our life now? This is crazy.” Everything just sort of falls apart and you focus on that child, because they’re really sick and it’s scary. (EM)
fear, hope	
	[My wife] met a woman who was 40 years old who had the disease since she was six. … that person’s disease had progressed actually quite a bit more than what we would ever hope that it progresses in our children, because of the lack of effective treatments. [Alluded wife exclaims: “Yeah. Huge.”], and the woman had a lot of crippling side effects at age 40. [Alluded wife confirms: “Oh yeah, unbelievable.”] However… Please finish the story. (NF) [Alluded wife continues:] Well, [this woman] she’s under, yeah, a lot of deformities, she’s going through a lot of surgeries – shoulder, wrist, finger, knees, ankles. It’s just, it’s an ongoing life… [of] treatment for her. Treatment for her life is going to be ongoing. …So I was so upset, I got my sister to phone the nurse here, to [hospital name], to find out the extent of what this looks like and where this could go, if this is a possibility for our children. And she said within her 20 years of working here and being the nurse on staff in this department, she has not seen… like maybe one case, maybe two, that severe, that severe [cases]. It’s very, very rare right now. (NM)
regret, shock, fear	
	We thought it was an ankle injury, and we did months and months with orthotics and everything else. And we have arthritis that runs in our extended family, and we never even thought of that [nervous laughs]. Oh, I’m getting emotional, but anyway, that’s exactly… So we weren’t prepared thinking it was arthritis. (EM)
	But now that I look back, I should have put two and two together because my husband does have arthritis. Not since he was a child, but my husband has had two hip replacements and he is in his 30s. (EM)
	No, I was just going to say, when my son started showing signs, I was praying, you know, “No, please no.” I knew. (EF)
denial	
	So we’re still not… We don’t have a genetic marker, so we still don’t have a proper diagnosis exactly, but he’s been told at least that it’s just pain management at the moment, he’s not damaging his joints. (NM)
	I’m still in denial about it though quite a bit… I mean I’m here and I want to be here for the knowledge for my daughter and for ourselves to apply to my daughter, but I am in very deep denial about her having arthritis. (NF)
anxiety, fear	
	Even my wife asked, “Well, like what if we come here this day and then like a week later, she has symptoms but we’re not coming back to you for two months and the damage [due to unnoticed inflammation of the eyes] is already done?” So basically [this] is what you do, get in a dark room or whatever and get a flashlight and just as long as their pupils dilate like they should… (laughs) [Another father adds: “It’s the proper medical way to do it.”] Yeah. And that I can take care of it in the basement with a flashlight. (everyone laughs) But that’s just if you’re really worried about it in the three months or whatever. I’ll trust the doctor for that. I’m just kidding. (laughs) It feeds into my denial though. “Yeah, you’re okay.” (everyone laughs) “Forget about it. I’m not worried about it.” (NF)
During Times of Disease Control	
fear, hope	
	Yeah. It’s a scary diagnosis to have, but there is hope with the treatment. (NF)
hope, gratitude, relief	
	[Daughter] got diagnosed when she was two. She’s almost seven… and, I don’t know, she… It works for her. The methotrexate is the best thing that came along for her, out of everything we have tried, and she’s been on it for three years … (EM)
fear, anxiety, frustration, fatigue	
	Well, it’s so overwhelming. It’s so overwhelming… You know, their immune suppressants are serious drugs, you know? What’s going to happen to my child if I put her on methotrexate for seven years? (change in voice intonation) What’s going to happen? You know, what’s the future going to look like for her? So it can have devastating consequences, the medications. And plus, the eye drops sometimes… We were on eye drops for seven years, every single day, sometimes every hour. (change in voice intonation) That’s a lot to take on. (EM)
anxiety, fear	
	I always worry about her being around sick people. She’s immune suppressed. I’m like, “Okay, has anyone in your house had a cold? Do you have ear infections?” Anything. I don’t want my daughter around sick people. When she goes to ballet and I see a kid, they’re coughing, I’m like, “Really? You’re sending your kid here sick? Thanks.” So I get really upset and emotionally charged when I see sick people. “You should be home. You’re going to make my kid sick. I could end up in emerg[encies], in the hospital for a month. It’s happened. (EM)
At Times of Increasing or Ongoing Symptoms	
sorrow, admiration, sympathy	
	It’s just tough for these kids. You know, it’s like he says like, “Why me?” you know? (crying) So it’s just… (gasp) Ah! It is what it is, but you know in some ways (gasp) – it’s interesting – it’s made him a stronger person. He’s really taking responsibility for his own health. And you know it’s early for a kid to do that. Like he’s growing up fast. And he’s… He’s meditating now. He’s probably the most popular kid in the school because he genuinely connects with people in a meaningful way. So he’s… (brief pause) It’s probably tougher on me right now than it is on him. (gasp) (NF)
sorrow, sympathy	
	She goes to bed on her own, you know, I don’t have to ask her; she just goes to the couch. …. Yesterday, we went boating. We were inner tubing, and she said, “Okay, I’ve had enough,” then she went to sleep in the boat. Or, in the middle of the afternoon, “Mommy, I’m going to go lie down.” But for a three or four-year-old to say, “Mommy, I’m going to go lie down,” to me, that’s a sign. You know, I… It’s just not normal for a child, three or four years old, or two, to put herself down for a nap. That’s a [sign], to me, that’s a sign that, hmmm… She doesn’t say that anything’s wrong, but it’s not normal for a child. (sigh) (EM)
frustration, indignation	
	Because one time at the nursery, my daughter was having a flare-up, and they called me urgently, “Come pick up your daughter, she’s being a princess, she’s throwing a… temper tantrum.” Seriously, my daughter is throwing a temper tantrum? That’s not like her. “Okay, fine.” She got home, she went to bed. That’s not normal. I went to [hospital’s name], and tests confirmed that she was having a flare-up. “Keep her at home; we’ll provide treatment.” And when I spoke with the director again, she told me, “No, I mean, that may have been part of it, but she was also having a bit of a princess tantrum.” Okay, fine, “If that’s your mentality, I’m certainly not going to argue about it with you.” I’m not going to argue; it’s not worth it. When people close the door, it’s just not worth it anymore. It doesn’t really matter what you have to say; their minds are made up and that’s that. (EF)
	Human beings are like that. We see someone who has no hair because of chemo treatments, and we’re sympathetic. But you know, I have an aunt with fibromyalgia, and that’s what she said too, at work. She stopped working, but chronic diseases, where there’s nothing [visible]. It’s like having an extreme backache, … and you tell your partner, “I can’t do it anymore, my back!” But it’s invisible. It’s the same thing with our children, without [outwardly visible illness signs] (EM)
frustration, sorrow	
	It’s huge. It affects my career. I have to take so much time off. Or they’re like, “Really? At [hospital name]?” … We were living here for six weeks one time. I’m like, I can’t go to work. There’s nothing I can do. Nobody really understands [at work]. You know, your boss, your peers, they’re sort of like, “Okay.” But really going through it, it’s terrible. (EM)
Frustration	
	Because I also had something to do [to convince child of taking medication]. We started with methotrexate in pill form, until her body realized it, and then just drinking her medication made her vomit. So we had to change to injections. But she’s had blood taken so often that she’s developed a phobia of needles. (EF)
Throughout the Course of the Illness	
powerlessness, anxiety	
	You don’t know what’s going to happen. It’s sort of living through the course of the disease that you sort of find your path and get comfortable within the system and all the health care providers that you deal with. I think over time, I’ve become more comfortable. (speed emphasis) Not really, because… (laughs) You can digest it, you research it, you read about it. You have all the information, but it’s still a rollercoaster ride for like eight years now. (EM)

*JIA* juvenile idiopathic arthritis, *EM* experienced mother, *EF* experienced father, *NM* novice mother, *NF* novice father

### At and shortly after diagnosis

Parents recalled the time of diagnosis as a shattering experience with strong feelings of shock, disbelief, and fear. Among novice parents these feelings were very strong, but these feelings were also vividly recalled by parents even more than a decade after diagnosis. Some parents strongly associated arthritis with the elderly, adding to their disbelief about their child’s diagnosis. Despite the potential of reaching longstanding remission in some types of JIA, parents expressed deep fears regarding the incurable or chronic nature of the disease, which was compounded by their limited knowledge about JIA at the time and the uncertainty about what to expect*.* At times, the fear was partially fuelled by their perceptions of arthritic physical deformity. They expressed fear and sorrow, but also tried to be hopeful about how their lives and children’s futures would be affected by the disease.

Additionally, experienced parents often felt blindsided by the diagnosis of JIA, especially those who had previous personal or family history with rheumatic diseases. Their previous experience with arthritis did very little to help them suspect and accept this diagnosis in their child.

Parents expressed varying degrees of denial as they attempted to come to terms with the diagnosis. A few novice parents still doubted the diagnosis even though they had agreed to participate in a study session for parents of children with JIA. Both novice and experienced parents humorously recounted some illogical things they did or beliefs they had at the time, and blamed these on their denial.

### During times of disease control

Later in the disease course, particularly at times of good disease control, parents felt less overwhelmed and better equipped to process information regarding their child’s illness. The news that effective treatments were available came as a great relief. At times of relative disease quiescence, parents predominantly felt gratitude, sometimes towards health care providers, as well as hope that administered treatments would continue to be effective.

These periods, however, were sometimes mixed with fatigue and frustration about the continuing need for treatment, as well as with anxiety regarding potential treatment side-effects. Parents were often fearful about the possibility of flares.

### At times of increasing or ongoing symptoms

During times of increasing or ongoing symptoms, many parents experienced a mix of admiration, sympathy, frustration, and sorrow. Parents felt admiration and sympathy towards their children for the way they courageously handled the disease and their efforts to carry on with their lives. Parents, however, simultaneously experienced sorrow and frustration about the disease making their child sit out of regular daily activities and feel pain.

During times of ongoing uncontrolled symptoms, some parents experienced frustration and indignation towards school teachers, their child’s peers, or family members, who doubted the veracity or extent of their child’s complaints and care needs. These feelings were sometimes also experienced towards individuals who did not seem to understand the demands of caring for a chronically-ill child, and the effects this had on parents’ careers. Parents were also frustrated about the need for increasing or changing treatments, with new potential side-effects.

### Throughout the course of the illness

Some emotions were not predominantly associated with specific disease periods, but rather formed a pervasive backdrop to the parental experience of caring for a child with JIA. These included an underlying anxiety and a sense of powerlessness, related to the long-term burden of managing an uncertain and time-consuming disease. Parents expressed a sense of strain through repeated phrases like: “lifelong,” “rest of your life,” “long road,” “long process,” “tiring,” “rollercoaster,” “ups and downs,” “life changing,” or “time consuming.” There was a sense that their lives and those of their children had been forever altered. On the positive side, parents expressed deep solidarity and companionship with their children. This was indicated by their speaking of their children’s experiences in plural expressions like: “when *we* were first diagnosed,” “*we* had a flare once,” or “that’s kind of how *our* diagnosis came about.”

### A recurring rollercoaster ride

**Fig. 1:**
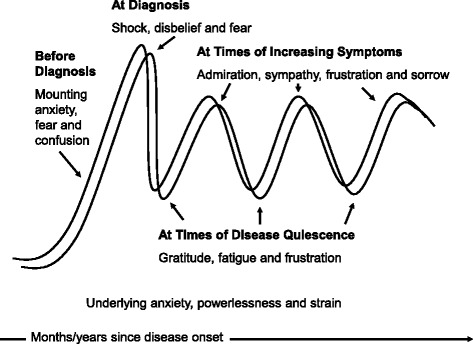
The affective journeys of parents of children with JIA may be described as recurring rollercoaster rides made of ups and downs with mixed negative and positive emotions

The overarching metaphor of a rollercoaster ride was mentioned by parents themselves on several occasions (see Fig. 1). This metaphor and the time periods described in this study emerged from the data as pertinent to synthesize the parents’ affective journeys. In other words, parents’ affective journeys resembled rollercoaster rides made of recurring, intensely felt emotional ups and downs, shaped by the uncertainty, variability, and disabilities associated with JIA.

On the whole, the emotional fluctuations, or ups and downs of parents’ rollercoaster rides, did not have a clear final resolution and were constantly changing even years after the diagnosis. While novice parents reported intense fear and disbelief early on, experienced parents reported that they continued to feel those emotions at other points during the disease course. Likewise, experienced parents reported mixed feelings of sorrow and admiration for their children, as did novice parents. Overall, the emotional experiences of novice and experienced parents were similar. The primary difference was that experienced parents had progressed further along in the trajectory of the disease, being exposed to a wider array of disease-related issues.

Lastly, we did not find clear differences in the emotional experiences of parents according to their own gender or the gender of their child. While we carried out sessions in English and French, similar emotional journeys were related in both languages. There were no major differences between the emotional experiences of parents of children who received medical care in one city or the other.

## Discussion

In this article we analyzed focus group audio-recordings, verbatim transcripts, and reciprocal interview reports from 23 parents of Canadian children with JIA. We found that mothers and fathers experienced complex mixed emotions that made the course of their children’s disease feel like a recurring emotional rollercoaster ride: a long-term, persisting affective journey of ups and downs associated with the unpredictable nature of the disease and the episodic physical and social disabilities associated with JIA. Emotions were not simple and sequential, but rather complex and overlapping. Unlike existing models suggesting discrete emotional stages and clear-cut emotional resolution reached once and for all [28], the parents of children with JIA in our study experienced sinuous emotional journeys of repeated ups and downs throughout their children’s disease..

Qualitative studies on parental emotional experiences have focused primarily on childhood cancer [16, 31, 32], with some studies devoted to asthma [33], cystic fibrosis [34], diabetes [35],congenital heart disease [36], anorexia [13], cleft palate [14], or autism [15]. Clinical quantitative research, mostly in the form of surveys, has reported the experiences [37], views [38], and preferences [39] of parents of children affected with JIA. However, little qualitative research has addressed their emotional experiences. Two significant exceptions are the studies of McNeill [19, 40] and Pelaez-Ballestas and colleagues [41].

McNeill explored the emotional experiences and gender identity constructions of fathers of children with JIA in Toronto, Canada. He found that despite prevailing assumptions of fathers’ lack of involvement in disease management, they were deeply affected emotionally by their child’s disease, and that JIA became a catalyst for meaningful, loving, and caring involvement with their child. Our findings confirm that fathers, and we add mothers, are profoundly emotionally affected by their child’s disease. McNeill found that fathers experienced some positive emotions, including maintaining an optimistic outlook of the disease, and being grateful for the opportunity that JIA had given them to be closer to their children. In our study, we also found positive emotions, including being grateful that effective JIA treatments existed, trusting they would work well on their child and admiration towards their child’s coping with the disease.

Pelaez-Ballestas and co-authors interviewed parents of children with JIA in Mexico City, Mexico. Similarly to our findings, they found that parents expressed strong mixed emotions, including concern about physical disabilities, inability to accept the diagnosis, guilt and despair, belittlement from doctors and/or spouses, and lingering denial. Pelaez-Ballestas *et al.* proposed the metaphor of a parental pilgrimage (emerging from their data) to convey parents’ descriptions of long, never-ending trajectories of intense suffering that resembled religious journeys, as they sought to come to terms with their children’s disease and get proper medical care. The notion of a pilgrimage has similarities and differences with our secular metaphor of a recurring rollercoaster ride. While a religious pilgrimage emphasizes the difficulties and sufferings parents face and their need to have faith, a rollercoaster ride accentuates the ups and downs, and the need to be prepared for the emotional curve or loop that comes next. Both metaphors, however, convey well the lengthy, sinuous, and textured affective journeys of mothers and fathers as they deal with their children’s JIA.

The emotional journeys parents recounted in the present study cannot easily fit into distinct or exclusive emotional categories. Their journeys have many “ins and outs,” as one father in our study put it, during which they are confronted with multiple and often contrasting emotions. Bowes and co-authors [35], have advanced a similar argument in the context of parenting a child with diabetes. Similarly, complex nuanced emotional experiences have been documented among parents of children with congenital heart defects, who remain hopeful as they struggle to understand and prepare for their child’s condition [36]. Parents of children with cleft palate also reported a mixture of delight, confusion, and distress during diagnosis and treatment [14]. Ambivalent – this is both positive and negative – emotional experiences have also been reported by parents of children with cancer, who welcome their children’s early hospital discharge after diagnosis with concern as well as with joy [31], or who experience confusion, relief, and fear after the end of treatment [32].

Our study and the studies cited above suggest that models of neatly ordered, initially negative emotional phases ending in positive resolution, likely inspired by Kübler-Ross’s five-stage model of loss and grief [28], do not convey the multifaceted emotional journeys experienced by parents of children with chronic illnesses. Parents in the present study described complex affective journeys with no clear resolution that could not be fully anticipated, and were akin to the ups, downs, and turns of a rollercoaster ride.

Correlations between patient outcomes and parental experiences have been demonstrated for JIA [8, 9] and other childhood chronic diseases [13–16, 42]. Although it seems logical that improving emotional support for parents may improve clinical and psychosocial outcomes for children, definitive proof in randomized trials of psychological interventions for parents reminds elusive [43]. Two cohort studies, using surveys of children with JIA and their parents, have shown an impact of parental distress on the child’s quality of life [44, 45] and one of them also showed an impact of social support on the child’s quality of life [45]. Awareness of the complex emotional journeys of parents of children with JIA may guide healthcare providers in offering better support for parents, and this in turn may result in better patient outcomes.

### Strengths and limitations

The study sessions of this study were facilitated by non-medical professionals previously unknown to the parents. This enabled parents to feel more comfortable in sharing their stories candidly and freely. The reciprocal interviews allowed for one-to-one exchanges with other parents and provided parents with an opportunity for direct input to the information that was analysed. The interdisciplinary composition of our research team, with backgrounds in medicine, nursing, psychology and anthropology added depth to the analytical discussions during the study.

The main limitation of our study is that it is a secondary analysis of qualitative data collected for a different primary purpose. We could not schedule additional sessions or interviews to follow-up and confirm our findings and insights. We recruited parents in two Canadian cities, and even though the described emotions were similar, we cannot assume that parents’ affective journeys are equivalent in other cities or territories. It is possible that experienced parents reported a selective view of their earlier felt emotions filtered through the experiences they had had during the period between disease onset and this research’s study sessions. Nevertheless, the reports of their initial emotional experiences were strikingly similar to those of novice parents.

There is of course a subjective component in identifying emotions at second hand; in fact sometimes it can be difficult for a person to clearly identify their own emotions. For this reason, our coding of emotions cannot be considered absolute or incontestable, but rather a reasonable emotion label given after discussion by a group of researchers considering original recordings and contextual circumstances. Since fluency in English or French, and willingness to participate in a group interview were pre-requisites for parents’ participation in the study, it is possible that the emotional experiences of parents with less education and less mastering of language (e.g. recent immigrants) are under-represented in our study*.* Two other possible limitations relate to generalizability of our findings and social desirability bias. It is possible that emotional experiences are different in other countries or cultures. This may explain some of the differences between our study and the one by Pelaez-Ballestas et al.[41]. It is also possible that given our focus group methodology, parents curtailed some of their emotional expressions in consideration of the group, even if the one-on-one interviews helped establish an accepting environment.

### Implications for research and clinical practice

We suggest that similar qualitative research should be conducted in other geographical locations, to confirm findings and better define the impact of sociocultural contextual factors on the emotional journeys of parents of children with JIA. Scholars applying a gender analysis to JIA [19, 40], and to other chronic illnesses [34, 42], have reported significant differences in the emotional responses of fathers and mothers. In future studies, gender differences could be addressed by holding separate sessions for mothers or fathers. Additionally, future projects could further explore the influence of the type of JIA on parents’ affective experiences. It would be important to focus more closely on the examination of parental emotional journeys in relation to JIA categories that present distinctive features, such as systemic JIA. Rheumatic autoimmune diseases affect mainly girls and women [46], and another area of scrutiny is the role of the ill-child’s age and gender in mediating parental emotional experiences [34]. It would also be important to examine the role of siblings, grandparents, and other family members in these affective journeys [42, 47]. In our study, similar emotions were reported by both experienced and novice parents. Future studies may assess in more detail how emotions change over time in order to confirm our findings and evaluate whether differences in reported emotions are merely a matter of degree. This could be done by carrying out a longitudinal qualitative study, following a cohort of parents over a period of time with repeated interviews or by asking them to keep a diary.

Although preliminary, our findings help delineate some basic suggestions for clinical practice. When preparing to meet parents during a clinic visit, care providers may pause and reflect on what point in their affective journey the parents may be, as this will aid them in tailoring their communications accordingly. Parents may benefit from being reassured during the initial visits that strong emotions that feel like fluctuating rollercoaster rides are expected and normal. Employing the metaphor of a recurring rollercoaster ride as a descriptive concept may be helpful in peer counselling or resource groups for parents. Health care providers in training may benefit from an overview of the recurring emotions experienced by parents and enumeration of the resources available to support parents through the ups and downs of the journey. Lastly, it would be important to reassure parents that strong emotions may be experienced even many years after diagnosis, and this does not mean that they are failing in adjusting to their child’s chronic disease.

## Conclusions

The parents of children with JIA included in this study reported complex mixed positive and negative emotions with ups and downs that felt like a recurring rollercoaster ride.  Healthcare providers caring for children with JIA should be aware of these complex emotional experiences in order to better support parents and help inprove JIA outcomes.

## Abbreviations

EF: experienced father
EM: experienced mother
JIA: juvenile idiopathic arthritis
NF: novice father
NM: novice mother
